# The influence of socioeconomic factors on the densities of high-value cross-border species, the African elephant

**DOI:** 10.7717/peerj.2581

**Published:** 2016-10-27

**Authors:** Sarah-Anne Jeanetta Selier, Rob Slotow, Enrico Di Minin

**Affiliations:** 1 Biodiversity Assessment and Monitoring, South African National Biodiversity Institute, Silverton, South Africa; 2 Amarula Elephant Research Programme, School of Life Sciences, University of KwaZulu-Natal, Durban, KwaZulu-Natal, South Africa; 3 Department of Genetics, Evolution and Environment, University College London, University of London, London, United Kingdom; 4 Finnish Centre of Excellence in Metapopulation Biology, Department of Biosciences, University of Helsinki, Helsinki, Finland; 5 Department of Geosciences and Geography, University of Helsinki, Helsinki, Finland

**Keywords:** Governance, Transboundary, Elephant, Distribution models, Biodiversity conservation

## Abstract

Unprecedented poaching levels triggered by demand for ivory in Far East Asia are threatening the persistence of African elephant *Loxodonta africana*. Southern African countries make an important contribution to elephant conservation and could soon become the last stronghold of elephant conservation in Africa. While the ecological factors affecting elephant distribution and densities have extensively been accounted for, there is a need to understand which socioeconomic factors affect elephant numbers in order to prevent conflict over limited space and resources with humans. We used elephant count data from aerial surveys for seven years in a generalized linear model, which accounted for temporal correlation, to investigate the effect of six socioeconomic and ecological variables on the number of elephant at the country level in the Greater Mapungubwe Transfrontier Conservation Area (GMTFCA). Important factors in predicting elephant numbers were the proportion of total land surface under cultivation, human population density and the number of tourists visiting the country. Specifically, elephant numbers were higher where the proportion of total land surface under cultivation was the lowest; where population density was the lowest and where tourist numbers had increased over the years. Our results confirm that human disturbance is affecting elephant numbers, but highlight that the benefits provided by ecotourism could help enhance elephant conservation. While future studies should include larger areas and more detailed data at the site level, we stress that the development of coordinated legislation and policies to improve land-use planning are needed to reduce the impact of increasing human populations and agriculture on elephant.

## Introduction

Growing human populations and rural poverty in Africa have led to an increasing demand for agricultural land (Krug, 2001). Between 1970 and 2005, wildlife abundance in African protected areas declined by 50% (Craigie et al., 2010), and many species’ ranges are now restricted to protected areas (Karanth, Nichols & Hines, 2010; Newmark, 2008). While protected areas are fundamental for biodiversity persistence in increasingly human-dominated landscapes (Baeza & Estades, 2010; Montesino Pouzols et al., 2014; Stokes et al., 2010), they are often too small to sustain viable populations of large mammals (Di Minin et al., 2013b; Graham et al., 2009; Packer et al., 2013), as they cannot meet the space requirements of wide-ranging or migratory species (Di Minin et al., 2013b; Graham et al., 2009; Stokes et al., 2010; Woodroffe & Ginsberg, 1998). Increasing human populations near protected area boundaries (Harcourt, Parks & Woodroffe, 2001) and elsewhere have further resulted in land-use conversions that prevents free movement of wildlife (Newmark, 2008; Wittemyer et al., 2008), embedding protected areas within a mosaic of different land uses such as agriculture, cattle grazing and mining (Chazdon et al., 2009; DeFries et al., 2007; Di Minin et al., 2013c). In many instances, this has led or could soon lead to increased human-wildlife conflict (Di Minin et al., 2016; Ogutu et al., 2011; Packer et al., 2013). Not only do those that live with dangerous species incur costs through human-wildlife conflict, but also governments incur the cost of protecting these species. For example the cost of anti-poaching measures as seen in the attempts to conserve and protect black (*Diceros bicornis*) and white rhinoceros (*Ceratotherium simum*) in South Africa (Di Minin et al., 2015).

Protected areas fall under a range of management strategies (Loveridge et al., 2007), and the resources allocated to, or generated within, these areas will directly relate to their ultimate success (Chase et al., 2016; Di Minin & Toivonen, 2015; Leader-Williams & Albon, 1988). There is, however, a marked underinvestment in state-protected areas, especially in developing countries. According to Balmford et al. (2002) the world spent approximately US$6.5 billion each year on the existing reserve network, yet half of this was spent in the United States alone. Effective African elephant (*Loxodonta africana*) conservation has been estimated to cost US$365–930/km^2^/year (Leader-Williams & Albon, 1988), while in unfenced reserves, such as in Kenya, the cost of protecting lion (*Panthera leo*) requires budgets in excess of US$2,000/km^2^ per annum (Packer et al., 2013). Within national conservation departments across Africa there is a shortage of manpower and ultimately resources (Leader-Williams & Albon, 1988; Selier & Di Minin, 2015), that may lead to the mismanagement of protected areas and a failure to protect species within these areas (Krug, 2001).

Illegal hunting of iconic species, such as elephant and rhino, has drastically increased over the past years in range countries with high poverty levels and bad governance (Bennett, 2015; Burn, Underwood & Blanc, 2011; Di Minin et al., 2015; Gandiwa et al., 2013; Maisels et al., 2013). Of the 12 countries in Africa estimated to have elephant populations larger than 15,000 individuals, eight are among the bottom 40% of the world’s most corrupt countries, and three are among the bottom 11% (Bennett, 2015). On the other hand, elephant range states in southern Africa have contributed positively to the conservation of elephant and hold more than 55% of the total elephant population on the continent (Blanc et al., 2007; Chase et al., 2016; CITES, IUCN & TRAFFIC, 2013). Outside of protected areas, pressure on wild animals is often higher, as elevated human densities around conservation areas can explain local species extinction (Brashares, Arcese & Sam, 2001; Chase et al., 2016). Effective protection is only achievable with the support of society at large, as success in protecting wild animals may depend not only on protection status or law enforcement efforts, but also on the desire of people to respect the law, to put the law into effect, and to tolerate or even admire wildlife (Stern et al., 2001). Thus, merely setting aside protected areas for the protection of species is not enough. In areas where elephant are present, human variables might better explain the present-day densities of elephant in Africa than ecological variables (de Boer et al., 2013; Selier, Slotow & Di Minin, 2015). It was further shown that anthropogenic activities, such as trophy hunting, within different management units forced elephants to trade-off between disturbance avoidance and good food and water availability (Selier, Slotow & Di Minin, 2015). Human factors are thus becoming dominant in determining the quality of the Earth’s ecosystems (Vitousek et al., 1997), and therefore need to be included in policy-relevant analyses (Selier, Slotow & Di Minin, 2015).

Southern Africa represents the stronghold of elephant conservation (Blanc et al., 2007; Chase et al., 2016). While the ecological factors affecting elephant distribution and numbers have extensively been accounted for, there is a need to understand which socioeconomic factors affect elephant numbers in those countries that have a positive contribution to elephant conservation. In this paper, we used the Greater Mapungubwe Transfrontier Conservation Area (GMTFCA) savanna elephant population (*Loxodonta africana*) as a case study to assess the effect of socioeconomic factors on the numbers of elephant within a cross-border landscape. We used the GMTFCA elephant population because, like many others (Chase & Griffin, 2009; Selier et al., 2014; van Aarde & Jackson, 2007), this population is transboundary, meaning that its range extends across international borders and beyond designated protected areas. This allowed us to test whether different socioeconomic factors, such as different levels of governance in different countries, are important in affecting numbers of a transfrontier elephant population. The general goal of this paper was to understand which socioeconomic and ecological factors affected elephant numbers in a transfrontier conservation landscape. The objectives were (i) to describe trends in the numbers of elephant over time; and (ii) to identify socioeconomic and ecological factors affecting the numbers of elephant.

## Methods

### Study area

**Figure 1:**
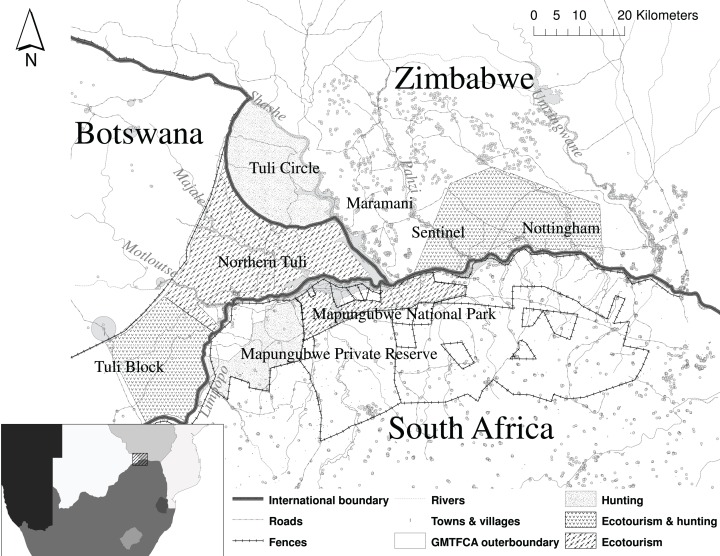
The Greater Mapungubwe Transfrontier conservation area and surrounding areas illustrating the borders between the three countries and the different sites within the countries used in the analysis.

This study was undertaken within the GMTFCA, in Botswana, South Africa and Zimbabwe (Fig. 1). The GMTFCA covers 3,650 km^2^ centered on the confluence of the Shashe and Limpopo Rivers. The region is semi-arid with low, unpredictable, rainfall (Harrison, 1984) that averaged 365 mm annually between 1966–2001 (Selier, 2007). Summer maximum temperatures can exceed 42 °C, while winter minimum temperatures can be as low as −5 °C (Mckenzie, 1990). The elephant population in the GMTFCA consists of approximately 1,224 ± 72.4 individuals (2000–2012) (Selier et al., 2014). Electric fences restrict the movement of elephant and other wildlife in certain sections. These fences extend along the western boundary of the Northern Tuli Game Reserve (NTGR), the northern boundary of the Tuli Block and along the Limpopo River on the South African side, with a gap in the fence known as the Vhembe gap around the confluence of the Limpopo and Shashe rivers (Fig. 1) (Selier et al., 2014).

The study area is characterized by a human-dominated landscape with a range of land use and management practices (Fig. 1) (Selier, Slotow & Di Minin, 2015). Land use, and ownership within and surrounding the GMTFCA, are diverse, and include contractual partners, private and communal landowners, land claimants, private tourism operations, game farms and subsistence and commercial farmers (Greater Mapungubwe Transfrontier Conservation Area TTC, 2011). The following sites were included in the study: the NTGR, and Tuli Block in Botswana, Tuli Safari Area, Maramani and Nottingham Estate and Sentinel Ranch complex in Zimbabwe and Mapungubwe National Park and Mapungubwe Private Nature Reserve in South Africa (Fig. 1). Several commercial operations operate within the current boundaries of the GMTFCA, all of which use, either for ecotourism or trophy hunting, this single cross-border elephant population that can move freely between the three countries. Ecotourism is the main economic driver within the area at present (Evans, 2010), but several operations rely on a combination of trophy hunting and ecotourism.

### Statistical analysis

We used a generalized linear model with Poisson distribution and a log-link function to examine the socioeconomic and ecological drivers of elephant numbers within the GMTFCA. The models were fit with autoregressive error structure to correct for temporal correlation using the *geepack* package (Halekoh, Højsgaard & Yan, 2006) in R v. 3.1.1 (R Development Core Team, 2014). The GMTFCA was divided into 3 different regions, one for each country (Botswana, South Africa and Zimbabwe) (Table 1). Elephant numbers within the GMTFCA per country per year (2000, 2001, 2004, 2007, 2008, 2010 and 2012) were used as the response variable. A total of six socioeconomic and ecological variables were used as covariates in the generalized linear model. All covariates were fitted as fixed effects–i.e. with constant regression coefficients across countries. Specifically, we determined the magnitude and direction of beta coefficients for each independent variable.

**Table 1:** Socioeconomic and ecological variables included in the generalized linear models with country included as a fixed effect to determine the variables that best explain elephant densities in the Greater Mapungubwe transfrontier conservation area.

Variable	Data description	Source
Enhanced vegetation index (EVI)	Forage availability at end of the dry season, raster, continuous data	CSIR-Meraka Institute 2011, 8-day composites; http://wamis.meraka.org.za/products/long-term-time-series
Agri	Proportion of total land surface under cultivation	http://data.worldbank.org/indicator
CPI	Corruption perception index (CPI score)	http://www.transparency.org/country
Human densities	People per km^2^	http://data.worldbank.org/indicator
Rural population growth rate	For people living in rural areas as defined by national statistical offices	http://data.worldbank.org/indicator/SP.RUR.TOTL.ZG
International tourism, number of arrivals	Number of tourists who travel to a country other than that in which they have their usual residence	http://data.worldbank.org/indicator/ST.INT.ARVL

Count data of elephant within the GMTFCA were obtained from total aerial counts conducted within the study area at the end of the dry season (July–September) over the period 2000–2012 (Selier, 2012; Selier, Slotow & Di Minin, 2015). Three fixed-wing aircrafts, flying 1 km transects, were used to count the study area simultaneously and the same method was used during all counts.

We were guided in the choice of candidate covariates by the aims of the analysis, in particular to enable characterization of countries and variables that were used in similar analyses (e.g. de Boer et al., 2013; Burn, Underwood & Blanc, 2011; Maisels et al., 2013). While regional data would be better to use, the data is simply not available for all three countries. Previous studies (e.g. de Boer et al., 2013; Burn, Underwood & Blanc, 2011; Maisels et al., 2013) followed a similar approach. After a correlation analysis using the cor() function in R 2.15.2 (R Development Core Team, 2012), with a cut-off of r = 0.80, we retained the six variables with the greatest explanatory effect on elephant numbers that were not strongly correlated (Franklin & Miller, 2009). The final explanatory variables used are summarized in Table 2.

**Table 2:** Beta coefficients of predictors of elephant numbers within the Greater Mapungubwe transfrontier conservation area.

	Beta	SE	Z	P-value	
(Intercept)	11.736	4.189	7.850	0.005	**
Forage availability	−0.412	0.823	0.250	0.616	
Corruption perception index	−0.554	1.706	0.110	0.745	
Land under cultivation	−5.276	0.619	72.730	0.000	***
Human density	−2.159	0.672	10.340	0.001	**
Rural population growth	−0.106	0.189	0.320	0.574	
Tourists visiting/year	0.289	0.088	10.740	0.001	**

**Note:**

Significance codes: 0 (***) 0.001 (**) 0.01 (*).

Food availability is a key ecological driver affecting elephant distribution (Chamaillé-Jammes et al., 2008; Ngene et al., 2009). Elephant are bulk feeders and thus occur in lower numbers in areas with lower plant biomass (Olff, Ritchie & Prins, 2002). We used the Enhanced Vegetation Index (EVI) as a measure of vegetation productivity, and thus the amount of forage available to elephant (Pettorelli et al., 2005; Young, Ferreira & van Aarde, 2009). The EVI data were downloaded for the period January 2000 to December 2012 (Table 1). The EVI time series was produced from the NASA 500 m, 8-day, BRDF-corrected, surface reflectance data (MCD43A4) (CSIR-Meraka Institute 2011). The log-transformed geometric mean of the 8-day composites for the end of each dry season of each of the count years were calculated with a grid cell size of 536.7 × 536.7 m, and used as a measure of the vegetation productivity per site per count year. The end of dry season was classified as the 8-day composite preceding the date of first 20 mm of rainfall with follow up rain within two weeks and overlapped with the respective aerial count dates.

Human densities are negatively correlated with elephant numbers (de Boer et al., 2013; Selier, Slotow & Di Minin, 2015; van Aarde & Jackson, 2007). The number of people per km^2^ within each country was thus included as a variable that may influence elephant numbers (Table 1). The proportion of the total land area under cultivation within each country also reflects human presence and may be used as a proxy for land fragmentation and the proportion of people that may be impacted on by wildlife through human-wildlife conflict (Abensperg-Traun, 2009). Country or regional policies, level of corruption and the capacity of a country to successfully implement policies further influence the level of protection provided. We therefore included the Corruption Perceptions Index (CPI) from Transparency International (http://www.transparency.org/) as an index of the level of corruption for each country as predictor variables (Table 1). We included CPI because it was extensively used in previous studies (Burn, Underwood & Blanc, 2011; de Boer et al., 2013; Smith et al., 2003). Rural population growth per country was included as a predictor variable because of its link to poverty. Higher numbers of people dependent on natural resources may lead to an increase in conflict between humans and elephant for these resources and a subsequent decline in elephant numbers (Wittemyer et al., 2008). On the other hand, the number of tourists visiting a country may increase the number of elephants in a country due to the benefits acquired through ecotourism on a country level (Krüger, 2005; Lindsey et al., 2005).

Values for human density, CPI, EVI and number of eco tourists visiting the country per year were not uniformly distributed and were log-transformed (Franklin & Miller, 2009).

## Results

According to the generalized linear model (Table 2), the best predictors for elephant densities were proportion of land under cultivation, human density and number of tourists visiting the country (Table 2). The coefficient for proportion of land under cultivation had–as expected a negative sign, indicating that an increase in cultivated land was expected to result in a decrease in elephant numbers (Table 2). The coefficient of human density also had a negative sign, indicating that an increase in human density was expected to result in a decrease in elephant numbers (Table 2). The coefficient of number of tourists visiting, instead, had a positive sign, indicating that an increase in the number of tourists was expected to result in an increase in elephant numbers (Table 2).

## Discussion

In this study, we used a generalized linear model to investigate the effect of socioeconomic and ecological variables on the numbers of elephant at the country level within the GMTFCA. We found that the proportion of land under cultivation, human density and number of tourists visiting were important in predicting elephant numbers. Particularly, elephant numbers were higher where the proportion of total land surface under cultivation was the lowest; where population density was the lowest and where tourist numbers had increased over the years. While future studies should include more countries and more detailed data at the site level, we stress the importance of common legislation and land use planning and enhanced benefit sharing with local people to enhance the persistence of elephants in a transboundary landscape.

The effective protection and conservation of high value species does not solely rely on increasing the size of protected areas or improving ecological conditions, but through considering the local socioeconomic conditions within the area (Adams et al., 2004; Burn, Underwood & Blanc, 2011; de Boer et al., 2013). This study showed that high human densities, the proportion of land under cultivation and tourist numbers are important factors predicting the abundance of elephant in those countries that positively contributed to elephant conservation (Blanc et al., 2007; de Boer et al., 2013). On one side, elephant numbers correlated negatively with encroachment of local human populations and agriculture, implying that these are having negative impacts on elephant numbers. On the other hand, increasing numbers of ecotourists visiting protected areas are having beneficial effects on the number of elephants. This is the big African challenge on how to more adequately reward locals for sharing the same landscape with elephants. Where, potentially, ecotourism benefits are shared with local communities, elephant numbers are increasing. Projected increases on human densities are expected to, and frequently have significant, negative impacts on biodiversity, such as illegal timber and mineral extraction (Curran et al., 2004). In absence of these and other benefits, increased human densities on the edges of protected areas may lead to an increase in human-wildlife conflict (Di Minin et al., 2016; Packer et al., 2013; Wittemyer et al., 2008), species extinctions (Brashares, Arcese & Sam, 2001; Packer et al., 2013; Woodroffe & Ginsberg, 1998), over-hunting (Brashares et al., 2004; Gandiwa et al., 2013), increased fire frequency (Kodandapani, Cochrane & Sukumar, 2004), and increased fragmentation of the landscape restricting elephant movements (Di Minin et al., 2013b; Selier, Slotow & Di Minin, 2015; Woodroffe & Ginsberg, 1998).

An increase in the proportion of land under cultivation will also negatively influence elephant numbers. Land use changes, such as an increase in cultivated land, reduce the size of natural ecosystems, and increase fragmentation of the landscape restricting the movement of wide-ranging species (Di Minin et al., 2013b; Selier, Slotow & Di Minin, 2015; Woodroffe & Ginsberg, 1998). In addition, agricultural expansion can isolate protected areas from their surrounding landscapes, leading to an island effect where no or limited connectivity exists between protected areas (Butchart et al., 2010; DeFries et al., 2007; Yackulic, Sanderson & Uriarte, 2011). Hoare & Du Toit (1999) showed that when agriculturally transformed land becomes spatially dominant over natural woodland elephants disappear from the system. In this human-dominated landscape, the size and connectivity of the remaining patches of elephant habitat will determine whether or not elephants remain as residents or move away (Hoare & Du Toit, 1999). Increased fragmentation of the landscape restricting the movement of wide-ranging species could further lead to increased human-wildlife conflict. Conflicting land use practices (crop farming) draw elephant and other conflict species towards community areas, primarily during periods of low natural food availability, thereby creating an ecological trap (Chiyo et al., 2005; Hoare & Du Toit, 1999; Nyhus & Tilson, 2004). Therefore, coordinated land use planning to maintain protected areas of sufficient size and maintain connectivity between protected areas will be required to maintain elephant in the study area and potentially limit human-elephant conflict. A lack of coordinated land use planning between range states may not only have implications for biodiversity in general, but also socioeconomic implications for tourism operations relying on the presence of these species.

Ecotourism has become a powerful tool for the conservation of threatened species worldwide by providing political support for conservation and generating economic benefits across all land tenures (Balmford et al., 2015; Krüger, 2005; Lindsey et al., 2005). Specifically the presence of charismatic megafauna is a major component in attracting eco tourists (Di Minin et al., 2013a; Krüger, 2005). Our results support this finding with higher elephant numbers predicted in countries where tourism numbers have increased over years. Ecotourism can further facilitate the restoration of elephant populations through the establishment of new populations in areas where elephants have previously been extirpated. In South Africa, the potential economic benefit derived from elephant through ecotourism has led to the re-introduction of approximately 800 elephants into more than 58 reserves (Slotow et al., 2005). A recent study has further shown that elephants prefer areas where ecotourism is the main activity compared to other land uses where anthropogenic disturbances force elephant to trade-off between good food and water availability and avoidance of human disturbances (Selier, Slotow & Di Minin, 2015).

The economic impact (real or perceived) of wildlife has a strong influence upon people’s attitudes towards conservation (Blignaut, De Wit & Barnes, 2008; Lindsey, Roulet & Romanach, 2007; Prins & Grootenhuis, 2000). Since communal lands comprise a large fraction of rural Africa (up to 500% more than state-managed forest reserves and national parks) (Alden Wily, 2011), economic incentives to communities that promote or at a minimum tolerate living with wildlife is an important solution to promote participation of local communities in biodiversity conservation efforts and improved enforcement (Child et al., 2012; Di Minin, Leader-Williams & Bradshaw, 2016; Naidoo et al., 2016). Thus, where funds derived from ecotourism are captured and appropriately distributed it has the potential to improve the livelihoods of local communities and their attitudes towards conservation leading to lower levels of human-elephant conflict. Finally, an alternative explanation to the higher number of elephants in Botswana compared to South Africa and Zimbabwe could potentially be the slow dispersal of elephant from the source population within the NTGR east and southwards into South Africa and Zimbabwe.

Where populations are transboundary, the joint management of elephant on a population level is imperative for their continued persistence in a human-dominated landscape (Linnell, Salvatori & Boitani, 2008; Trouwborst, 2015). This will require the development of coordinated legislation and policies to improve land-use planning (Chapron et al., 2014; Montesino Pouzols et al., 2014; Trouwborst, 2015), the development of multi-use zones around protected areas (Wittemyer et al., 2008), and conservation corridors to link current protected areas between range countries (van Aarde & Jackson, 2007). In order to maximize conservation benefits and to alleviate the impacts of future human growth and land-use changes on wildlife action should be taken quickly. Benefits generated from ecotourism and trophy hunting might help retain elephants and other species that co-exist with them (Di Minin, Leader-Williams & Bradshaw, 2016; Di Minin et al., 2013c). Effective protection of source populations in a well-connected system of protected areas that buffers them from anthropogenic threats remains the key action to ensure the future persistence of wide-ranging species, such as elephant, in the developing world (Di Minin et al., 2013b; Di Minin & Toivonen, 2015).

## Supplemental Information

10.7717/peerj.2581/supp-1 Supplemental Information 1 Great elephant count survey data.

## Additional Information and Declarations

### Data Deposition

The following information was supplied regarding data availability: The data was provided as part of the recent publication: Chase MJ, Schlossberg S, Griffin CR, Bouch� PJC, Djene SW, Elkan PW, Ferreira S, Grossman F, Kohi EM, Landen K, Omondi P, Peltier A, Selier SAJ, Sutcliffe R. 2016. Continent-wide survey reveals massive decline in African savannah elephants. PeerJ 4:e2354 DOI 10.7717/peerj.2354 and is also uploaded as Supplemental Dataset Files.
